# Using Extract From the Stems and Leaves of Yizhi (*Alpiniae oxyphyllae)* as Feed Additive Increases Meat Quality and Intestinal Health in Ducks

**DOI:** 10.3389/fvets.2021.793698

**Published:** 2022-01-31

**Authors:** Fengjie Ji, Lihong Gu, Guang Rong, Chengjun Hu, Weiping Sun, Dingfa Wang, Weiqi Peng, Dajie Lin, Quanwei Liu, Hongzhi Wu, Haofu Dai, Hanlin Zhou, Tieshan Xu

**Affiliations:** ^1^Tropical Crops Genetic Resources Research Institute, Chinese Academy of Tropical Agricultural Sciences, Haikou, China; ^2^Institute of Animal Science and Veterinary Medicine, Hainan Academy of Agricultural Sciences, Haikou, China; ^3^Institute of Tropical Bioscience and Biotechnology, Chinese Academy of Tropical Agricultural Sciences, Haikou, China

**Keywords:** Yizhi (*Alpinia oxyphylla*), ducks, intestinal health, antibiotic alternatives, meat quality

## Abstract

Yizhi (*Alpiniae Oxyphyllae, A. oxyphylla*) has been widely used as an important traditional Chinese medicinal herb for centuries. Existing studies have shown that *A. oxyphylla* has numerous benefits in human and animal health. We hypothesized that extract from the stems and leaves of *A. oxyphylla* (AOE) as a feed additive may have positive effects on animal health and products. Thus, this study was conducted to evaluate the effects of AOE as a feed additive on growth performance, serum biochemical parameters, intestinal morphology, microbial composition, and meat quality in Jiaji ducks. A total of 240 Jiaji ducks of 42 days old (1675.8 ± 44.2 g, male: female ratio = 1:1) were blocked based on body weight and randomly allocated into four dietary treatments with three replicates that each had 20 duck individuals. The dietary treatments included: basal diet, control group (CK); basal diet supplementation with 30 mg/kg (Y1), 80 mg/kg (Y2), and 130 mg/kg (Y3) AOE, respectively, and lasted for 49 days. The results showed that average daily feed intake from day 42 to day 60 was decreased with the increasing level of AOE (*P* < 0.05). Compared with the CK group, the groups with AOE supplementation decreased serum LDL-C level (*P* < 0.05), the addition of 30 mg/kg AOE increased total amino acids, essential amino acids, branched-chain amino acids, nonessential amino acids, and umami taste amino acids (*P* < 0.05), but decreased selenium and zinc concentrations in breast muscle (*P* < 0.05). In addition, the supplementation of 30 or 130 mg/kg AOE significantly increased jejunal villus height (*P* < 0.05) and tended to increase the ratio of villus height to crypt depth in the jejunum (*P* = 0.092) compared to the CK group. Moreover, the addition of 30 mg/kg AOE showed a higher abundance of genus *unclassified Bacteroidales* and genus *unclassified Ruminococcaceae* than the CK group (*P* < 0.05). Therefore, dietary supplementation with 30 mg/kg AOE increased meat nutrition profile and flavor through promoting amino acid contents in breast muscle, as well as maintained intestine integrity and modulated the microbial composition. In conclusion, AOE as an antibiotic alternative displayed potential in maintaining intestinal health and improving meat quality.

## Introduction

Nowadays, adding antibiotics supplementation at the sub-therapeutic level to the diet of poultry as a growth promoter has been widely banned in many countries due to the increasing demand for safe animal food and the great concern regarding the spreading of antibiotic resistance. Jiaji duck, a Muscovy duck variety with good meat quality, has been raised for more than 200 years and has become one of the fourth most famous meals in the Hainan province (1). However, after the banning of antibiotics, intensive breeding of Jiaji duck has faced the great challenges of reduction in growth rate, risk for intestinal barrier dysfunction, susceptibility to infectious disease, and decline in meat quality and production profits. Therefore, it is particularly urgent to research green, safe, and efficient alternatives to antibiotics in feeding this duck variety.

Plant extracts have been proposed to serve as antibiotic alternatives due to their benefits for poultry production. For example, the mixture of phytoecdysteroids extracted from the juice of *Serratula coronata L*. was reported to increase the live weight, average daily live weight gain, and gutted carcass weight by 4.5%, 3.0–3.5%, and 7.1%, respectively in meat ducks (2). Dietary supplemented with 0.01% or 0.02% grape seed extract increased the growth performance and antioxidative capacity in Pekin ducks (3). *Tinospora cordifolia*, a traditional plant species in Indian Ayurveda medicine, yielded positive impacts on growth performance and meat quality in broilers (4). Resveratrol, an anti-inflammatory plant extract, yielded positive impacts on meat quality in Pekin duck through stimulation of intramuscular fat and flavor amino deposition and alteration of muscle fiber characteristics (5).

Yizhi (*Alpiniae Oxyphyllae, A. oxyphylla*) belongs to the Zingiberaceae family and grows in the tropical and subtropical regions of China (6). Fructus *A. oxyphylla* is an important traditional Chinese medicinal herb that has been widely used in the treatment of abdominal pain, and for its antidiarrheal, anti-polyuric, and anti-salivation qualities for centuries (7, 8). Existing studies showed that *A. oxyphylla* has numerous benefits including anti-tumor (9), anti-ulcer (10), antidiarrheal (11), antioxidant (12), anti-inflammatory (13), and neuroprotective (14) activities. Based on this evidence, we hypothesized that the extract from the stems and leaves of *A. oxyphylla* (AOE) might have potential as an antibiotic alternative to improve animal health and production. Therefore, this study was conducted to investigate the effects of dietary AOE on growth performance, meat quality, intestine development, and microbial composition in Jiaji ducks, to evaluate the potential of using AOE as a green feed additive that can improve the intestinal health and meat quality of Jiaji ducks.

## Materials and Methods

### Animals and Treatments

Jiaji ducks were obtained from Hainan Chuanwei Muscovy Duck Breeding Co., Ltd. A total of 480 Jiaji ducks at the age of 42 days old with an average initial body weight of 1675.8 ± 44.2 g (male: female ratio = 1:1) were blocked based on body weight. There were 6 replicates (pens) per treatment and 20 ducks per pen in a randomized complete block design. Dietary treatments included: (1) basal diet, control group (CK); (2) basal diet + 30 mg/kg AOE, Y1; (3) basal diet + 80 mg/kg AOE, Y2; (4) basal diet + 130 mg/kg AOE, Y3. The feeding experiment lasted for 49 days. The pens were equipped with feeder, drinker, and raised plastic floors. Diets were fed in pellet form and ducks were provided with water and feed *ad libitum* throughout the experiment. The basal diet (Table 1) was formulated to meet the Nutrient Requirement of Poultry (National Research Council, 1994). The body weight of each replicate was weighed at 42, 60, and 90 days of age, respectively. The feed intake per replicate was daily recorded. The average daily feed intake (ADFI), average daily gain (ADG), and ratio of feed intake to body weight gain (F:G) of ducks from each replicate were calculated accordingly.

**Table 1:** Composition and nutrition levels of the basal diet.

**Ingredients**	**Content (%)**	**Nutrient Levels**	**Content (%)**
Corn	70.53	ME (MJ/kg)	12.27
Soybean meal	23.50	Crude protein	16.79
Wheat bran	1.70	Crude fat	3.05
CaHPO_4_	1.37	Crude fiber	2.63
Limestone	0.90	Calcium	0.46
L-Lysine	0.14	Available P	0.35
DL-Methionine	0.18	L-Lysine	0.95
Premix^a^	1.68	Methionine	0.45
Total	100		

a *One kilogram of multiple vitamin premix contained: vitamin A, 50,000,000 IU; vitamin B1, 10,000 mg; vitamin B2, 20,000 mg; vitamin B6, 10,000 mg; vitamin B12, 5,000 mg; vitamin C, 4,000 mg; vitamin D, 1,000,000 IU; vitamin E, 60,000 IU; vitamin K3, 8,000 mg; folic acid, 2,500 mg; niacin, 80,000 mg; pantothenic acid, 30,000 mg; biotin, 2,200 mg; Cu, 5 g; Fe, 50 g; Zn, 55 g; Mn, 55 g; I, 0.3 g; Se, 0.22 g*.

### Plant Material and Preparation of Extract

The stems and leaves of *A. oxyphylla* were collected from the Tropical Botanical Garden in Danzhou City, Hainan province, China and were identified by Professor Haofu Dai. The plant tissues were air-dried at 60°C, and ground to powder with a stainless-steel blender and extracted referring to a method by Wang et al. (12). In brief, plant powder was extracted three times in a reflux condenser for 1.5 h each with 95% ethanol at 55/60°C. The solution was combined and filtered. Solvents were removed by using a rotary vacuum evaporator. Finally, the crude extract was condensed in a freezer-dryer, and brown powder was obtained.

### Sample Collection and Preparation

At the end of the trial, 2 ducks with a similar average body weight from each replicate were sacrificed for blood and tissue samples. The blood was collected from the wing vein into a sterile tube and stored at room temperature for 30 min, then centrifuged at 3000 × g for 10 min at 4°C. Serum was separated into Eppendorf tubes, and preserved at −80°C for further analysis. After bleeding, the birds were quickly excised in an aseptic operation to obtain tissue. The jejunum (the midpoint of the small intestine) and ileum (10 cm distal to the ileocecal junction) segments were fixed in 4% paraformaldehyde for analysis of intestinal morphology. Breast muscle samples were collected from the breast and then stored at −80°C. The cecum was separated and the fecal sample was collected into a 10 ml sterile tube and stored at −80°C for further analysis.

### Serum Biochemical Parameter

Serum biochemical indexes including total protein (TP), albumin (ALB), glucose (GLU), alanine aminotransferase (ALT), aspartate aminotransferase (AST), creatinine (CRE), urea, uric acid (UA), triglycerides (TG), total cholesterol (TC), high-density lipoprotein cholesterol (HDL-C), and low-density lipoprotein cholesterol (LDL-C) were determined *via* the biochemical analytical instrument PUZS-600B (Medical Equipment Co., Ltd., Beijing, China) using respective commercial assay kits.

### Measurement of Intestinal Morphology

Intestinal morphology was measured following a procedure reported in Xiong et al. (15): after being rinsed with physiological saline, proximal 2 cm segments were promptly fixed in 10% formalin. Then fixed samples were dehydrated in graded ethanol and embedded in paraffin. A 5-μm thickness section was cut using a microtome (RM2235; Leica; Germany), then stained with hematoxylin-eosin. Histological images were captured using a light microscope (Olympus Bx51, Japan). The villus height and crypt depth were determined by image analysis system Image-Pro Plus 6.0 software (Media Cybernetics, MD, USA).

### Cecum Feces Microflora 16S rDNA Sequencing

The DNA extraction, PCR amplification, 16S rDNA sequencing, and bioinformatics analysis were produced by the LC-Bio Technology Co., Ltd, Hang Zhou, Zhejiang Province, China. DNA Extraction and PCR Amplification: Microbial DNA was extracted from the cecal feces sample (*n* = 9 / group) using the E.Z.N.A. ®Stool DNA Kit (D4015, Omega, Inc., USA) according to the manufacturer's instructions. The total DNA was eluted in 50 μl of Elution buffer and stored at −80°C until measurement in the PCR by LC-Bio Technology Co., Ltd, Hang Zhou, Zhejiang Province, China. The V3-V4 region of the 16S rDNA gene was amplified using primers 341F (5′-CCTACGGGNGGCWGCAG-3′) and 805R (5′-GACTACHVGGGTATCTAATCC-3′). The PCR products were purified by AMPure XT beads (Beckman Coulter Genomics, Danvers, MA, USA) and quantified by Qubit (Invitrogen, USA). The amplicon pools were prepared for sequencing and the size and quantity of the amplicon library were assessed on Agilent 2100 Bioanalyzer (Agilent, USA) and with the Library Quantification Kit for Illumina (Kapa Biosciences, Woburn, MA, USA), respectively. The libraries were sequenced on NovaSeq PE250 platform.

Data Analyses of Microbial Communities: raw sequencing data were assembled and filtered using QIIME (Version 1.9.1) and the fqtrim (v0.94) software to obtain clean tags. Chimeric sequences were filtered using Vsearch software (v2.3.4). Feature tables and feature sequences were obtained after dereplication using DADA2. Alpha diversity was used to measure the complexity of species diversity and five different indices were calculated using QIIME2, including Chao1, observed species, Goods coverage, Shannon, and Simpson. Beta diversity was also calculated using QIIME2. The blast was used for sequence alignment, and the feature sequences were annotated with the SILVA database for each representative sequence. Other diagrams were implemented using the R package (v3.5.2).

### Meat Free Amino Acid Composition

The contents of free amino acid in breast muscle were analyzed by an amino acid analyzer (L-8900, Hitachi, Japan) as described in the previous study (16). Approximately 100 mg samples were dissolved using the water–methanol (1: 1, v/v) at 4°C for 30 min and centrifuged at 10 000 × g for 10 min. The supernatant was then filtered through glass wool and stored at −80°C until analysis. After centrifugation, soluble material was separated from insoluble material, and 40 ml of the supernatant was labeled with iTRAQ reagents (AA 45/32 kit; Applied Biosystems, Foster City, CA, USA) as recommended by the manufacturer and analyzed using an Applied Biosystems 3200 Q TRAP LC/MS/MS system equipped with an RP-C18 column (length of 150 mm, diameter of 4.6 mm and particle size of 5 mm). Amino acids in the samples were quantified based on known standards (Shimadzu Oceania, Pickering Laboratories, Mountain View, CA, USA) and their retention times using LC Solution ver. 1.22 SP1 software (Shimadzu, Kyoto, Japan).

### Meat Fatty Acid Composition

The fatty acid composition in breast muscle was analyzed by gas chromatography according to the previous method (16). Lipids were extracted from the meat samples using the chloroform methanol (1:1, v/v) procedure. Fatty acid methyl esters were prepared for GC determination using KOH/methanol. Fatty acid methyl esters were analyzed using an Agilent 6890N gas chromatographer equipped with a flame ionization detector (Agilent Technologies, Santa Clara, CA, USA). A CP-Sil 88 fused silica open tubular capillary column (100 m × 0.25 nm; Chrompack; Thermo Scientific, Shanghai, China) was used. The initial oven temperature was set at 45°C for 4 min and then increased to 175°C at 13°C/min, held at 175°C for 27 min, increased to 215°C at 4°C/min and then held at 215°C for 35 min. The injector and detector temperatures were set at 250°C. The carrier gas was hydrogen at a flow rate of 30 ml/min. The identification of individual fatty acid methyl esters was accomplished by the retention times of an authentic standard. The concentration of individual fatty acids was quantified according to the peak area and expressed as a percentage of total fatty acids.

### Meat Trace Elements Fe, Se, Zn

The iron (Fe), selenium (Se), zinc (Zn) concentration in breast muscle was determined according to the Chinese National Standard Method (GB 5009.268-2016). Grounded samples were subjected to microwave acid digestion as reported by Zhang et al. (17). Briefly, ~0.5 g of breast muscle samples were weighed and mixed with 8 ml of acid solution (HNO_3:_ H_2_O = 5: 95) in 50 ml digestion tubes, then kept at room temperature overnight. The lysate was heated in a microwave digestion system (CEM-MARSX®, CEM Corporation, Matthews, NC, USA), as follows: 120°C for 5 min, 150°C for 10 min, and 190°C for 20 min. After full decomposition, the digestion vessels were placed in an ultrasonic water bath for 5 min to evaporate gas, and the contents of the vessels were transferred into centrifuge tubes, and deionized water was added to fulfill the tubs to be 25 ml. And the solution was filtered through a 0.45 μm membrane, fully mixed for further analysis.

Instrumental analysis: Fe, Se, and Zn standard solution was used to construct standard curves. The Fe, Se, and Zn concentrations were detected using an Agilent 7,900 inductively coupled plasma mass spectrometry (ICP-MS) system (Agilent Technologies, Santa Clara, CA, USA). The ICP-MS operating parameters for as follows: RF power = 1550 W, plasma gas flow = 15 L/min, carrier gas flow = 0.8 L/min, auxiliary = 0.40 L/min, helium gas flow = 4 L/min, sampling depth = 10 mm, Spray chamber type = Babington digestion/Concentric Nebulizer. The internal standard, Germanium (Ge), was added to each sample according to the manufacturer's instruction to correct for sample losses due to volatility and evaporation.

### Statistical Analysis

The results were presented as means with respective standard error of the mean (SEM). The data were analyzed by one-way ANOVA procedures of SPSS v. 22.0 software (SPSS Inc., Chicago, IL, USA), followed by Duncan's multiple comparison tests. Significant differences between means were indicated by *P* < 0.05, and a trend toward significance was indicated by *P* < 0.10. The relative species abundances and overall composition (at phyla and genera levels) of intestinal microbial communities were analyzed using the Kruskal-Wallis test. Linear discriminant analysis coupled with effect size (LEfSe) was used to identify different taxa microbes among lines using default parameters. Spearman's correlation coefficient was used to assess the relationships between the environmental factors and the relative abundances of microbial genera.

## Results

### Growth Performance

As shown in Table 2, dietary treatments did not affect (*P* > 0.05) final body weight, F: G, ADG. During days 42–60, ADFI in the Y3 group was decreased compared with the CK or Y1 groups (*P* < 0.05). During days 61–90 or 42–90, ducks in the Y3 group also had low feed intake, but did not significantly differ from other groups (*P* > 0.05).

**Table 2:** Effects of dietary AOE on growth performance in ducks.

**Items**	**CK**	**Y1**	**Y2**	**Y3**	**SEM**	***P-*value**
D42 BW (g)	1670.00	1720.00	1643.33	1670.00	12.76	0.192
D60 BW (g)	2576.67	2625.83	2586.67	2539.17	21.65	0.631
D90 BW (g)	3322.73	3332.54	3258.25	3300.37	15.27	0.355
**Day 42–60**						
ADG (g/d)	50.37	50.32	52.41	48.29	0.83	0.423
ADFI (g/d)	152.87^ab^	156.09^a^	148.32^bc^	147.24^c^	1.27	0.015
F:G	3.03	3.12	2.83	3.05	0.05	0.142
**Day 61–90**						
ADG (g/d)	24.87	23.56	22.39	25.37	0.76	0.569
ADFI (g/d)	145.25	145.08	140.26	137.08	1.96	0.425
F:G	5.84	6.27	6.37	5.44	0.24	0.565
**Day 42–90**						
ADG (g/d)	34.43	33.60	33.64	33.97	0.34	0.849
ADFI (g/d)	148.11	149.21	143.28	140.89	1.59	0.200
F:G	4.31	4.44	4.26	4.15	0.05	0.320

a,b,c *In the same row, values with different letters mean significant difference (P < 0.05)*.

*ADG, average daily gain; ADFI, average daily feed intake; F:G, Feed intake / body weight gain. Y1: basal diet with 30 mg/kg AOE; Y2: basal diet with 80 mg/kg AOE; Y3: basal diet with 130 mg/kg AOE*.

### Serum Biochemical Parameter

**Figure 1:**
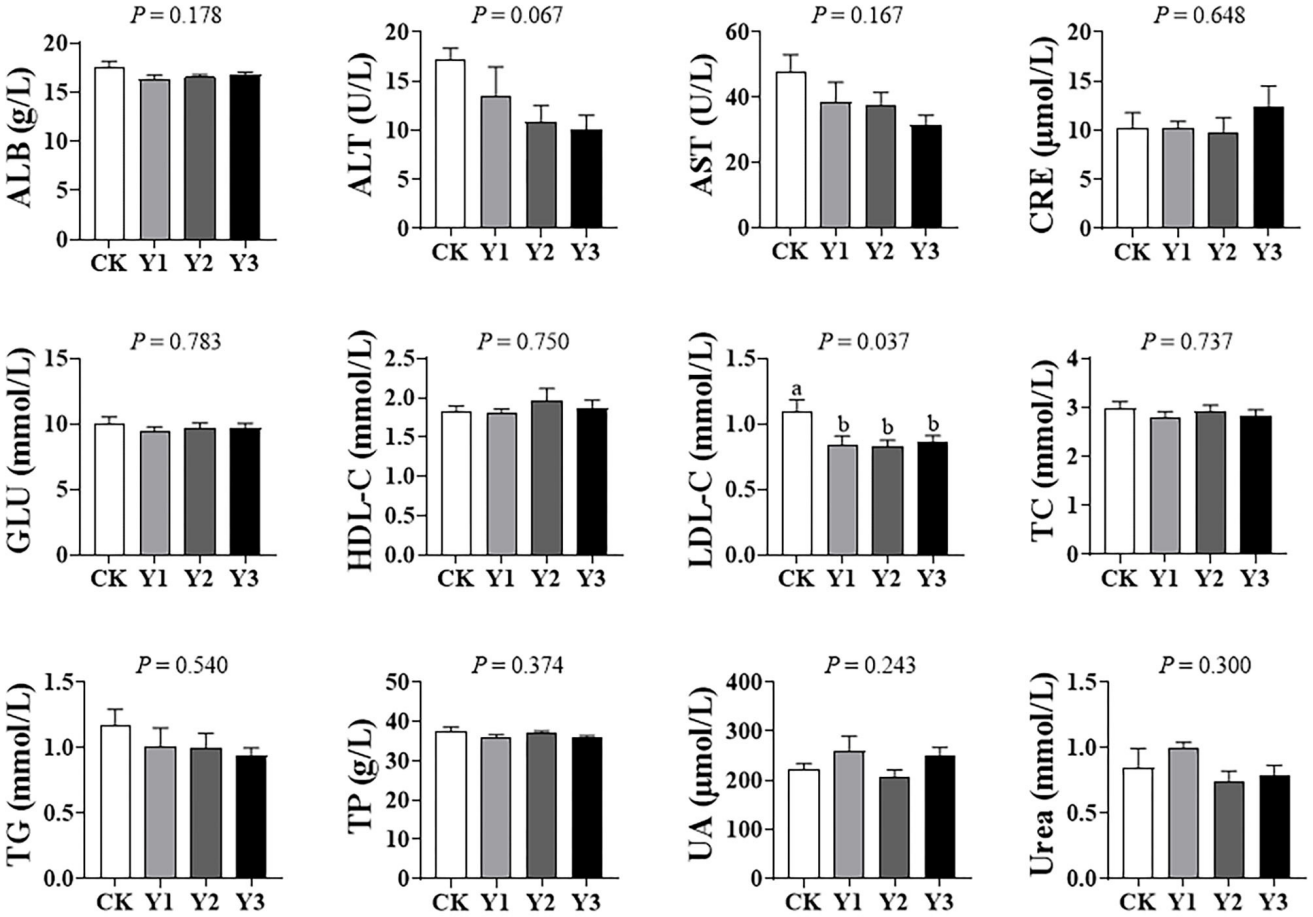
Effects of dietary AOE on serum biochemical parameter in ducks.

As shown in Figure 1, dietary AOE did not affect (*P* > 0.05) the levels of ALB, ALT, AST, CRE, GLU, HDL-C, TC, TG, TP, UA, and urea in serum. Compared with the CK group, the three groups supplementation with AOE were observed to decrease (*P* < 0.05) serum LDL-C.

### Intestinal Morphology

**Figure 2:**
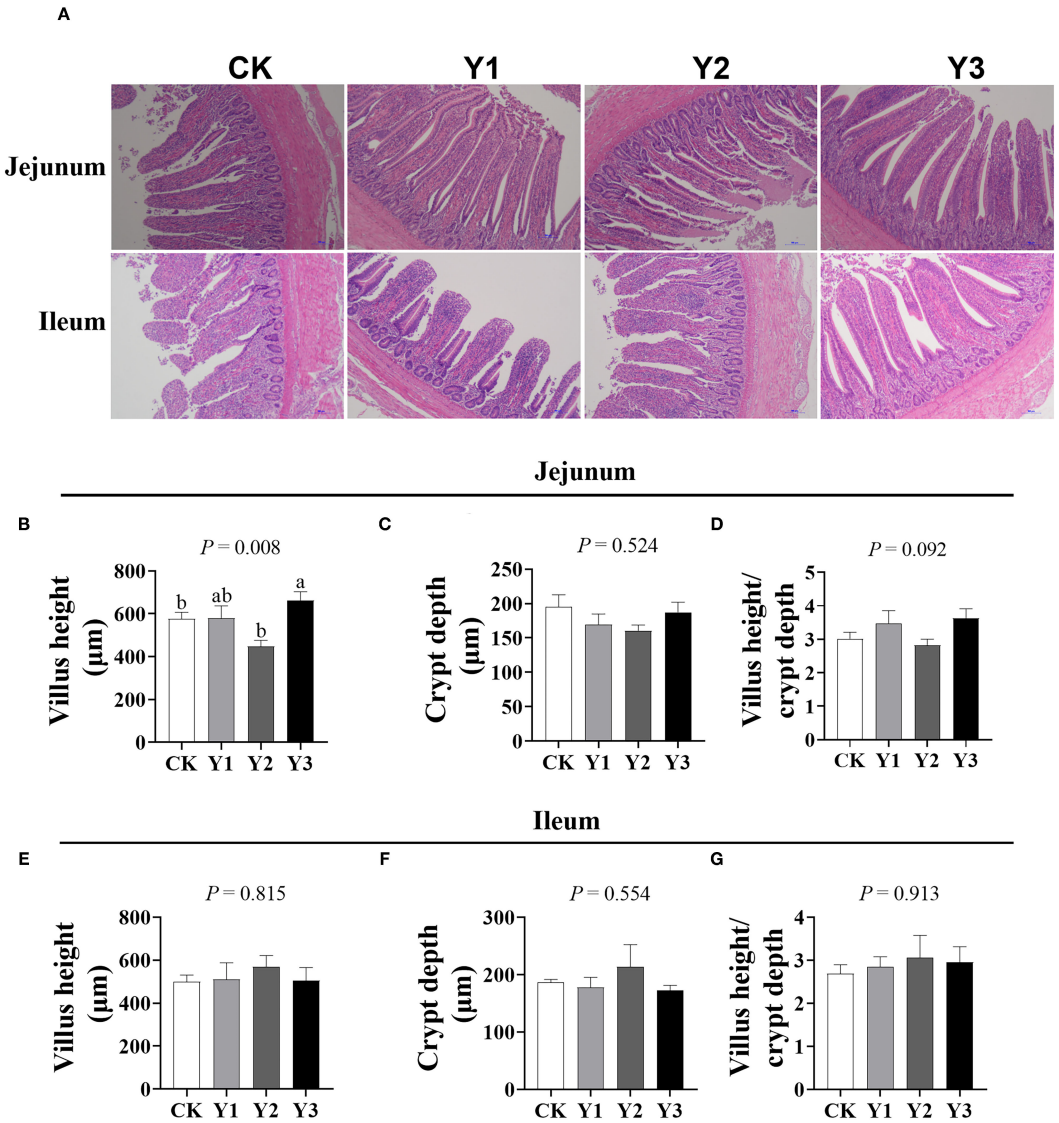
Effects of dietary AOE on intestinal morphology of ducks. **(A)** The images of the jejunum and ileum morphology, magnification 10 ×; Summarized data of villus height, crypt depth, and the ratio of villus height to crypt depth in jejunum **(B–D)** and ileum **(E–G)**.

As shown in Figure 2, the villus height in the jejunum was higher in Y1 and Y3 groups than that in the CK group (*P* < 0.05); in addition, the ratio of villus height to crypt depth in the jejunum in the Y1 and Y3 groups tended to increase (*P* = 0.092). There was no difference (*P* > 0.05) in villus height, crypt depth, and the ratio of villus height to crypt depth in the ileum among the four groups.

### Cecum Feces Microflora 16S rDNA Sequencing

**Figure 3:**
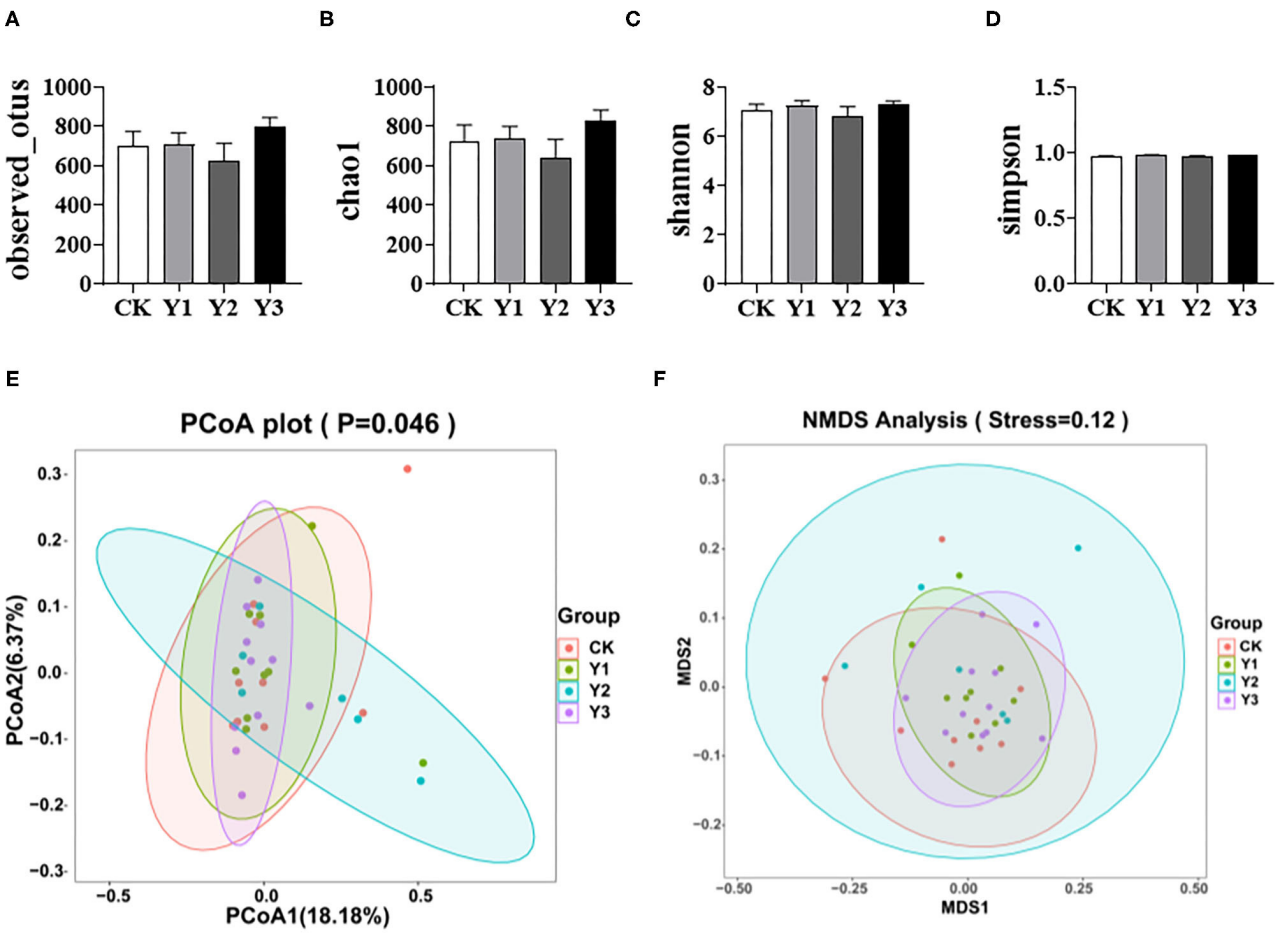
Effects of dietary AOE on alpha diversity and beta diversity of cecal bacterial community of ducks. **(A–D)** Alpha diversity including observed otus, chao1, shannon, and simpson indexes. **(E,F)** Beta diversity including Principal Coordinate Analysis (PCoA) and Non-metric multidimensional scaling (NMDS).

As shown in Figures 3A–D, dietary AOE had no effects on the observed otus, chao1, shannon, and simpson indexes of cecum microbiota (*P* > 0.05). As shown in Figures 3E,F, the Y2 group fed with 80 mg/kg AOE had higher (*P* < 0.05) PCoA and NMDS indexes than the CK group. In addition, the Y1 group showed similar PCoA and NMDS indexes to the CK group.

**Figure 4:**
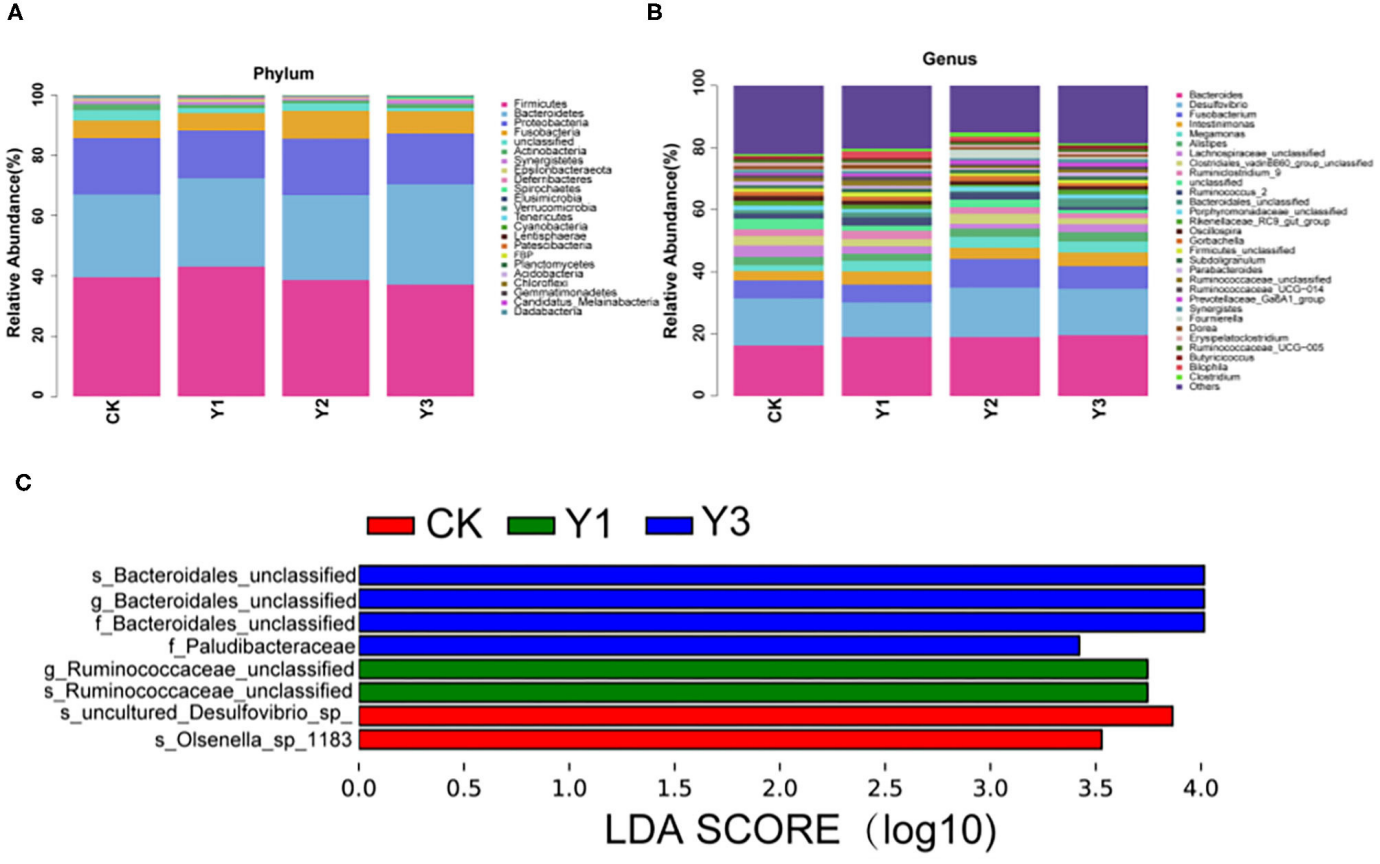
Effect of dietary AOE on microbial composition in cecum feces of ducks. **(A)** The abundance of the top 15 bacteria at phylum level. **(B)** The abundance of the top 15 bacteria at the genus level. **(C)** LEfSe Analysis. LDA, linear discriminant analysis.

As shown in Table 3 and Figure 4A, at the phylum level, the most dominant phyla among microbiota communities were *Firmicutes* (39.66%), *Bacteroidetes* (29.56%), *Proteobacteria* (17.55%), and *Fusobacteria* (7.04%), these microbiotas accounted for more than 93% of the total microbiota found in fecal samples. The abundances of *Firmicutes* were highest in the Y1 group. There was no difference in the microbiota communities at the phylum level among the four groups (*P* > 0.05). At the genus level (as showed in Table 4 and Figure 4B), the most dominant genra among microbiota communities were *Bacteroides, Desulfovibrio, Fusobacterium, Intestinimonas, Megamonas, Alistipes, unclassified Lachnospiraceae, Ruminiclostridium_9, Ruminococcus_2*.

**Table 3:** Effects of dietary AOE on phylum-level relative abundances of the cecal microbiota in ducks (%).

Phylum^*^	**CK**	**Y1**	**Y2**	**Y3**	***P*-value**
*p_Firmicutes*	39.63	43.16	38.68	37.18	0.24
*p_Bacteroidetes*	27.44	29.30	28.19	33.31	0.51
*p_Proteobacteria*	18.73	15.87	18.75	16.86	1.00
*p_Fusobacteria*	5.83	5.77	9.22	7.33	0.26
*p_unclassified*	3.41	1.62	2.49	1.10	0.24
*p_Actinobacteria*	2.02	1.14	0.94	1.32	0.30
*p_Synergistetes*	0.99	0.89	0.65	1.15	0.69
*p_Epsilonbacteraeota*	0.54	0.78	0.35	0.18	0.40
*p_Deferribacteres*	0.51	0.64	0.31	0.31	1.00
*p_Spirochaetes*	0.21	0.06	0.05	0.73	0.62
*p_Elusimicrobia*	0.16	0.10	0.07	0.21	0.31
*p_Verrucomicrobia*	0.09	0.32	0.07	0.04	0.91
*p_Tenericutes*	0.23	0.08	0.08	0.12	0.71
*p_Cyanobacteria*	0.12	0.15	0.13	0.10	0.64
*p_Lentisphaerae*	0.04	0.08	0.03	0.04	0.58

* *Top 15 most abundant species are listed. The relative species abundances of gut microbial communities were analyzed using the Kruskal–Wallis test, n = 9*.

**Table 4:** Effects of dietary AOE on genus-level relative abundances of the cecal microbiota in ducks (%).

Genus^*^	**CK**	**Y1**	**Y2**	**Y3**	***P*-value**
*g_Bacteroides*	16.29	19.01	19.00	19.61	0.83
*g_Desulfovibrio*	15.16	11.15	15.96	14.93	0.68
*g_Fusobacterium*	5.81	5.77	9.22	7.33	0.26
*g_Intestinimonas*	2.99	4.22	3.59	4.36	0.52
*g_Megamonas*	1.92	3.42	3.57	3.56	0.72
*g_Alistipes*	2.65	2.27	2.61	2.98	0.60
*g_Lachnospiraceae_unclassified*	3.70	2.39	1.55	2.55	0.46
*g_Clostridiales_vadinBB60_group_unclassified*	2.98	2.30	3.20	1.95	0.75
*g_Ruminiclostridium_9*	2.23	2.72	2.13	1.59	0.19
*g_unclassified*	3.41	1.62	2.49	1.10	0.24
*g_Ruminococcus_2*	1.65	2.71	2.27	1.04	0.64
*g_Bacteroidales_unclassified*	1.01^ab^	1.55^ab^	0.43^b^	2.58^a^	0.03
*g_Porphyromonadaceae_unclassified*	1.61	1.10	1.46	1.34	0.18
*g_Rikenellaceae_RC9_gut_group*	1.51	1.38	0.61	1.54	0.25
*g_Oscillospira*	1.57	1.30	1.15	1.11	0.23
*g_Gorbachella*	1.31	1.30	1.62	0.93	0.22
*g_Firmicutes_unclassified*	1.07	1.41	0.84	1.13	0.43
*g_Subdoligranulum*	0.98	1.21	1.15	1.12	0.68
*g_Parabacteroides*	1.33	0.94	0.57	1.24	0.22
*g_Ruminococcaceae_unclassified*	1.02^b^	1.63^a^	0.55^b^	0.89^b^	0.01
*g_Ruminococcaceae_UCG-014*	0.97	1.31	0.65	1.06	0.64
*g_Prevotellaceae_Ga6A1_group*	0.62	0.96	1.34	1.21	0.41

* *Genus with proportion under 1.00% are not listed. The relative species abundances of gut microbial communities were analyzed using the Kruskal–Wallis test, n = 9*.

a,b,c *In the same row, values with different letters mean significant difference (P < 0.05)*.

At the genus level, the Y1 and Y3 groups were higher than the CK group in the abundance of *unclassified Bacteroidales* (*P* < 0.05). Moreover, the Y1 group showed a higher abundance of *unclassified Ruminococcaceae* (*P* < 0.05) than the CK group. Microbial compositions between groups were further analyzed using the LEfSe (showed in Figure 4C). The LEfSe results showed that the genus *unclassified Ruminococcus* and species *unclassified Ruminococcus* were significantly enriched in the Y1 group. Family *unclassified Bacteroides*, genus *unclassified Bacteroides*, and species *unclassified Bacteroides* were significantly enriched in the Y3 group. Species *uncultured Desulfovibrio_sp*. and species *Olsenella_sp_1183* in the CK group were significantly enriched.

### Meat Free Amino Acid Composition

**Figure 5:**
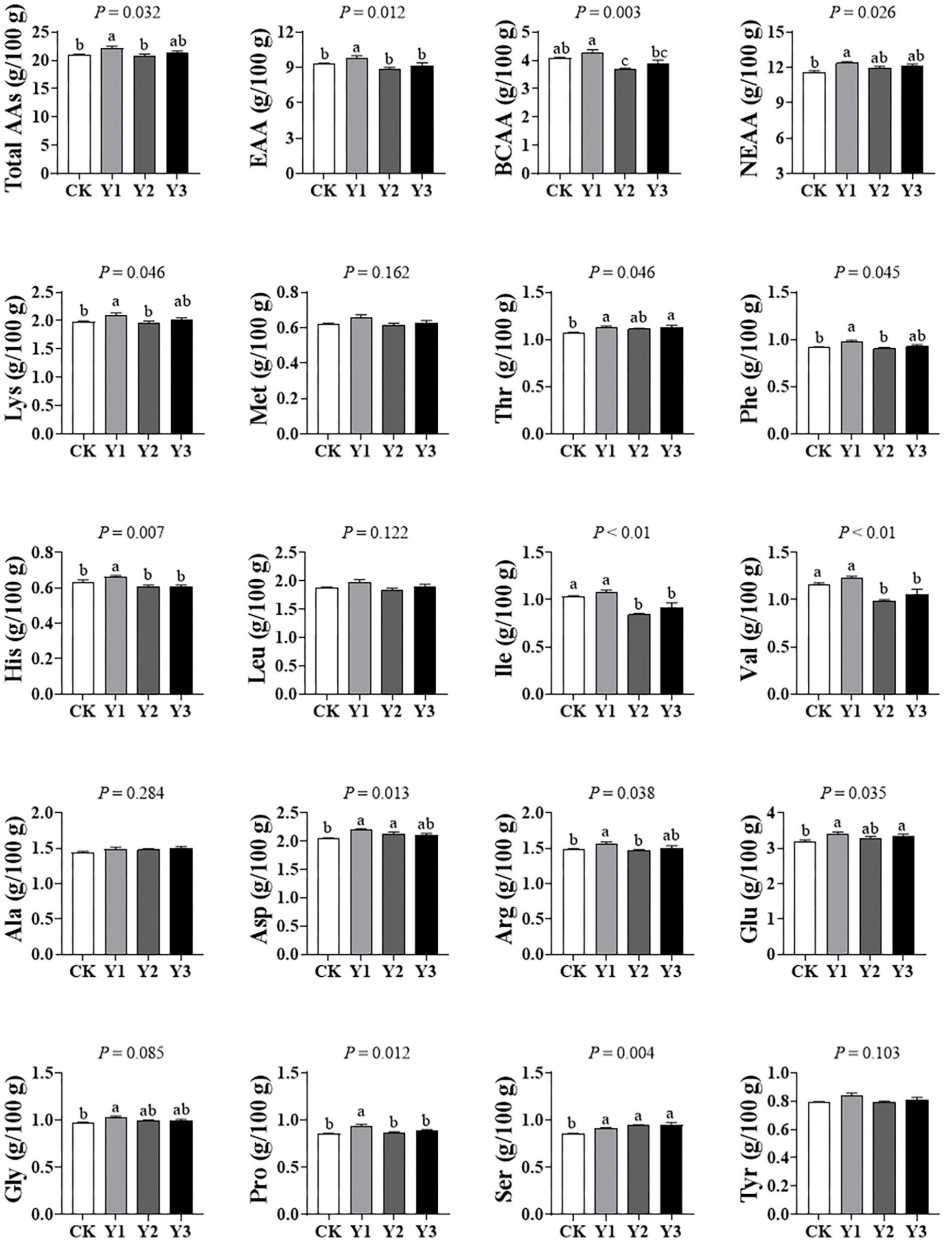
Effects of dietary AOE on meat free amino acid composition in breast muscle of Jiaji ducks. EAA = lysine (Lys) + methionine (Met) + threonine (Thr) + phenylalanine (Phe) + histidine (His) + leucine (Leu) + isoleucine (Ile) + valine (Val). BCAA = Leu+ Ile+Val. NEAA = alanine (Ala) + aspartate (Asp) + arginine (Arg) + glutamate (Glu) + glycine (Gly) + proline (Pro) + Serine (Ser) + tyrosine (Tyr).

As shown in Figure 5, the total amino acids (Total AAs), total essential amino acids (EAA), total branched-chain amino acids (BCAA), and total nonessential amino acids (NEAA) in breast muscle were highest in the Y1 group than other groups (*P* < 0.05). For lysine (Lys), methionine (Met), tryptophan (Tyr), threonine (Thr), phenylalanine (Phe), histidine (His), leucine (Leu), isoleucine (Ile), and valine (Val), they are included in EAA. For alanine (Ala), aspartate (Asp), arginine (Arg), glutamate (Glu), glycine (Gly), proline (Pro), Serine (Ser), tyrosine (Tyr), they are classified as NEAA. For Leu, Ile, and Val, belong to BCAA. In addition, essential Lys, His, Phe, and Thr in the duck meat of theY1 group was higher than in the CK group (*P* < 0.05). Essential His, Ile, Phe, and Val and nonessential Pro were decreased in the Y2 and Y3 groups compared with the Y1 group, suggesting that amino acid deposition in duck meat was reduced with AOE supplementation level increasing. For Arg, Asp, Glu, His, Phe, and Ser, most of them are referred to collectively as the flavor amino acid, they were also significantly increased in the Y1 group compared with the CK group (*P* < 0.05).

### Meat Fatty Acid Composition

As shown in Table 5, dietary AOE supplementation had no effects (*P* > 0.05) on the fatty acid composition in breast muscle, including C14:0, C16:0, C16:1, C18:0, C18:1n-9, C18:2n-6, C18:3n-6, C20:3n-6, C20:4, C24:0, C22:6ns, MUFA, PUFA SFA, and total fatty acid.

**Table 5:** Effects of dietary AOE on meat fatty acid composition in breast muscle of ducks.

**Items (g/100 g)**	**CK**	**Y1**	**Y2**	**Y3**	**SEM**	***P-*value**
C14:0	0.007	0.008	0.007	0.007	0.0004	0.946
C16:0	0.31	0.27	0.25	0.31	0.0161	0.457
C16:1	0.03	0.03	0.02	0.03	0.0018	0.370
C18:0	0.13	0.12	0.12	0.14	0.0060	0.517
C18:1n-9	0.44	0.36	0.39	0.42	0.029	0.794
C18:2n-6	0.26	0.23	0.23	0.28	0.0106	0.265
C18:3n-6	0.012	0.010	0.009	0.012	0.0006	0.125
C20:3n-6	0.005	0.005	0.005	0.006	0.0004	0.640
C20:4	0.090	0.092	0.084	0.104	0.0046	0.550
C22:6ns	0.007	0.008	0.007	0.009	0.0005	0.516
C24:0	0.02	0.02	0.02	0.03	0.0010	0.386
MUFA	0.473	0.385	0.410	0.453	0.031	0.777
PUFA	0.370	0.335	0.330	0.378	0.014	0.556
SFA	0.475	0.415	0.403	0.488	0.023	0.498
Total fatty acid	1.325	1.1425	1.1488	1.3525	0.0662	0.587

*SFA = C10:0 + C12:0 +C14:0 + C15:0 + C16:0 + C17:0 + C18:0 + C20:0 + C23:0*.

*MUFA = C14:1 + C16:1 + C17:1 + C18:1n9t + C18:1n9c + C20:1 + C24:1*.

*PUFA = C18:2n6t + C18:2n6c + C18:3n6 + C18:3n3 + C20:3n6 + C20:4n6 + C20:5n3 + C22:6n6*.

### Meat Trace Elements Fe, Se, Zn

**Figure 6:**
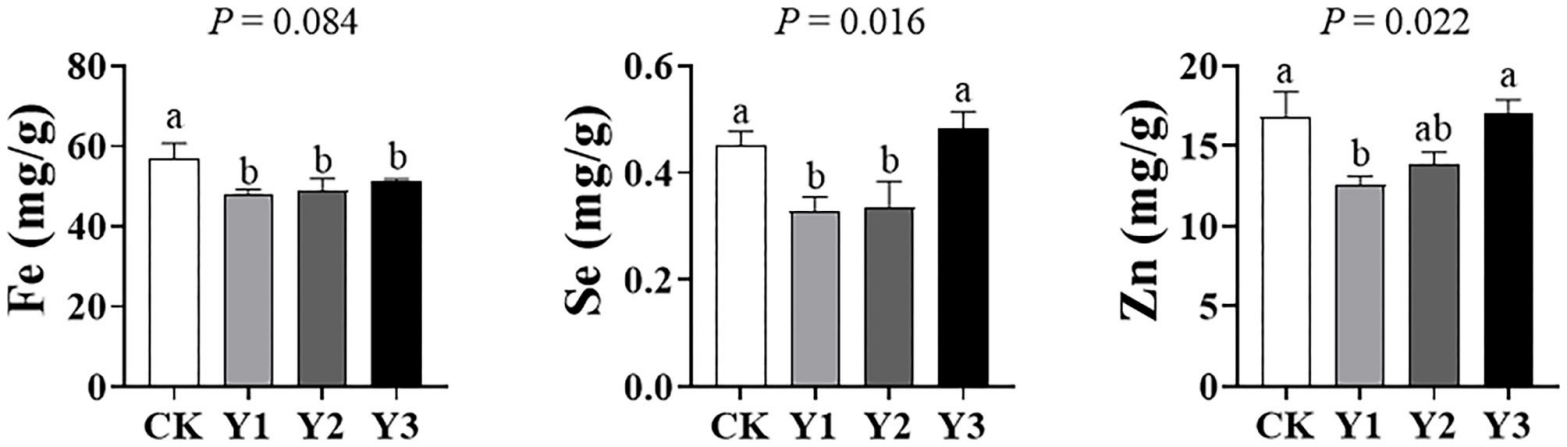
Effects of dietary AOE on meat trace elements in breast muscle of ducks.

As shown in Figure 6, the concentrations of Se and Zn were higher in the CK group (*P* < 0.05) than in the Y1 or Y2 group. In addition, the concentration of Fe in AOE groups tended to decrease (*P* = 0.084) compared with the CK group.

### Spearman's Correlation Analysis

**Figure 7:**
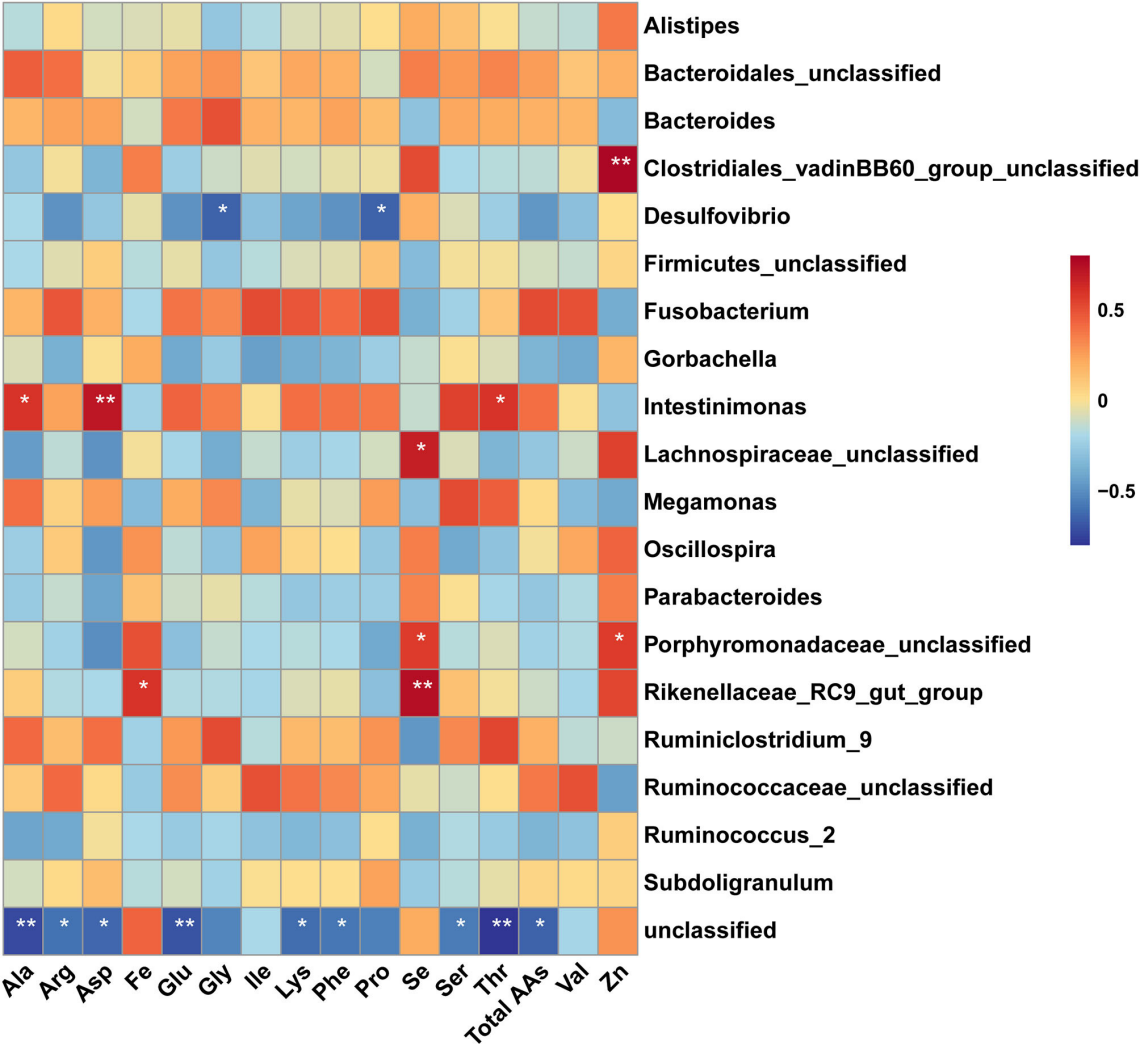
Spearman's correalation analysis of amino acid and dominant genus bacteria. The colors range from blue (negative correlation) to red (positive correlation). * Indicates significant difference (*P* < 0.05), ** indicates extremely significant difference (*P* < 0.01).

Spearman's correlation analysis of the dominant genus bacteria in the cecum and amino acids in the muscle was shown in Figure 7. The result showed that the abundances of the *unclassified Clostridiales_vadinBB60_group* and *unclassified Porphyromonadaceae* were positively correlated with the concentration of Zn in breast muscle (*P* < 0.05). The abundances of *unclassified Lachnospiraceae, unclassified Porphyromonadaceae*, and *Rikenellaceae_RC9_gut_group* were positively correlated with the concentration of Se in breast muscle (*P* < 0.05). Moreover, the abundance of *Rikenellaceae_RC9_gut_group* was positively correlated with the concentration of Fe in breast muscle (*P* < 0.05). The abundance of *Intestinimonas* had positively correlated the levels of Ala, Asp, and Thr in muscle (*P* < 0.05). While the abundance of *Desulfovibrio* was negatively correlated with the levels of Pro and Gly in muscle (*P* < 0.05). In addition, the abundance of genus unclassified bacteria was negatively correlated with the level of amino acid, including total amino acids, Phe, Lys, Thr, Ser, Glu, Asp, Arg, and Ala.

## Discussion

*A. oxyphylla* is an important traditional medicinal herb and plays an important role in animal health due to its anti-ulcer (10), antidiarrhea (11), antioxidant (12), and anti-inflammatory (13) properties. However, the effects of extract from the stem and leaf of *A. oxyphylla* (AOE) on the growth performance, serum biochemical parameter, meat quality, intestinal development, and microbial composition of ducks are still unclear. In this study, lower feed intake was observed in ducks fed with 130 mg/kg AOE diets, this may be explained by the slightly spicy nature of *A. oxyphylla*, but this needs to be confirmed. Many studies reported that various essential oils are beneficial in poultry performance (18, 19) but not all studies displayed the growth-promoting effect of plant essential oils in duck performance (20, 21). In line with previous results, our study found that dietary AOE had no effects on final body weight, F: G, and ADG of ducks. Conversely, in Pekin ducks, a study noted that dietary inclusion of 0.2% grape seed extract (anthocyanidins, catechins) could increase about 3.1% final body weight, and could improve F: G (3). The discrepancy might be explained by different plant sources and administered dosage.

To further investigate the effects of AOE on the health of ducks, we evaluated the serum biochemical parameter, which can help to assess the response of animals to various physiological, nutritional, and pathological scenarios (22). The increased level of AST, particularly ALT in serum is often accompanied by liver injury in mouse (23), while serum creatinine often indicates kidney damage (24). In the present study, we noted that dietary AOE supplementation had no effects on the levels of ALB, ALT, AST, CRE among the treatments, suggesting that AOE is safe for ducks without any damage to liver and kidney. Changes in the serum concentrations of TC, TG, and LDL-C can reflect dynamic lipid absorption and nutritional status in animals (25). The elevated serum levels of TC and LDL-C were more strongly associated with risk of cardiovascular disease (26). A 1% elevation in either TC or LDL-C increases the risk for Coronary heart disease by 2–3% (27). Moreover, cardiovascular disease was one of the leading causes of death in the Chinese. Therefore, in the present study, the decrease of serum LDL-C by AOE supplementation was of importance in the health of animal and human.

The intestinal epithelium barrier is constantly exposed to damage from luminal contents and pathogenic organisms. Efficient protection of the organism by this barrier relies on both eliminating pathogens (28) and the integrity of the epithelial sheet (29). The villus histology structure can reflect the integrity of the epithelium barrier. Given the increased ADFI in ducks fed with 30 mg/kg AOE, we suspected that dietary AOE might promote intestine development, which is the reason for our determination of the intestine morphology of ducks in this study. We found that ducks fed with 30 or 130 mg/kg AOE positively altered jejunal architecture, which suggested that this additive might protect small intestine integrity and maintain gut health. In line with our findings, previous investigations have shown that *A. oxyphylla* can promote the migration and proliferation of human adipose tissue-derived stromal cells (30, 31), which can help repair the gastrointestinal barrier.

The gut microbiota, the largest symbiotic ecosystem with the host, is consisted of trillions of microbes (32), which forms the intestinal microbiological barrier. Diet and dietary components have profound effects on the health and survival of the gut microbiota and are among the most important contributors to the alteration in bacterial flora (33). The comprehensive characterization of the microbial community in the intestine of ducks is vital in understanding the host healthy state and predicting the variations in microbiome-related to feed changes (34). The cecum has relatively lower oxygen partial pressure and decreased enzyme and bile salt concentrations that create conditions suitable for a variety of bacteria, thus, the majority of bacteria associated with avian species have been found in the cecum (35). The changes in intestine morphology suggest that AOE supplementation improved intestinal health. Therefore, we investigated the effects of dietary AOE on cecal microbial composition. *A. Oxyphylla* is widely used as traditional herbal medicine for the treatment of gut-associated diseases, including diarrhea, abdominal pain, intestinal disorders, and inflammatory conditions (8, 11, 13). A previous study has shown that *A. oxyphylla* can maintain body health in type 2 diabetes mellitus mice by modulating gut microbial composition (36). In the present study, the duck groups fed with AOE were higher in beta indexes than the CK group, indicating that dietary AOE increased microbial diversity. In this study, the microbial community in duck feces, *Firmicutes* (39.67%), *Bacteroidetes* (29.56%), and *Proteobacteria* (17.55%) were found to dominate, which was consistent with previous studies (37).

In the present study, *Bacteroides* (18.48%) were revealed to be the main genus in duck cecum, which was similar to the findings of several previous studies in ducks (34, 38–40). At the genus level, the Y1 group had a higher abundance of *unclassified Bacteroidales* and *unclassified Ruminococcaceae* than the CK group. The huge abundance of *unclassified Bacteroidales* may account for the fecal butyrate increment (41). And *unclassified Ruminococcaceae* strongly contributes to feed digestion (42, 43) and acetate production (44, 45). Acetate and butyrate as short chain fatty acids are a primary energy source for colonocytes and are known to strengthen the gut barrier function (46, 47). Therefore, dietary supplementation with 30 mg/kg AOE might maintain intestinal barrier function, by increasing the abundance of *unclassified Bacteroidales* and *Ruminococcaceae*.

Given the alteration in intestinal morphology and cecal microbial composition, the indexes of meat quality, including amino acid and trace elements were further analyzed. The amino acid profile is considered the most crucial nutritional property of meat. EAAs are not synthesized endogenously by metazoans and need to be supplemented to the diet, such as Lys, Met, Thr, and Phe. BCAAs, including Leu, Ile, and Val, constituting ~35% of EAAs in most mammals (48). BCAAs are building blocks for all life-forms, and exert vital roles in lipid and protein metabolism modulation (25), BCAAs are also responsible for energy homoeostasis, survival, growth, and immunity (49). Here, we observed that dietary supplementation with 30 mg/kg AOE significantly increased the contents of total amino acids, EAAs, and BCAAsin breast muscle, indicating that AOE displayed important roles in producing high-quality duck meat. It should be noted that amino acids can not only provide key nutritional value, but also significantly contribute to the taste and flavor of meat (50). For example, taste-active amino acids impart a sweet (Gly, Ala, Ser, Thr, Pro, Hyp), sour (Phe, Tyr, Ala), bitter (His, Arg, Ile, Leu, Lys, Phe, Val), and umami (Asp and Glu) taste on meat (51). In the present study, Asp and Glu, umami taste amino acids, were increased in AOE groups, suggesting that dietary supplementation with AOE also enhances the palatability of duck meat. This may be associated with the pleasant aroma of the *A. oxyphylla* plant. Therefore, AOE can demonstrate beneficial effects on duck meat, and its inclusion in the diet may allow Jiaji duck valuable protein sources for human.

Meat with iron, zinc, and selenium is of great importance to reduce the incidence of some diseases and accumulate as a part of beneficial effects on human health (52). In this study, dietary supplementation with 30 mg/kg AOE decreased the Zn and Se content in breast muscle, suggesting that supplementation of AOE decreased their accumulation in breast muscle. However, the mechanism needs to be further elucidated.

Furthermore, a Spearman correlation analysis was conducted to evaluate the relationship between environmental factors and the genus bacteria. The results showed that the abundance of *Intestinimonas* was positively correlated the level of sweet amino acid (Ala, Thr) and umami amino acid Asp in muscle, while the abundance of *Desulfovibrio* was negatively correlated with the level of the sweet amino acid such as Gly and Pro in muscle. *Intestinimonas* is a prevalent butyrate-producing species in the intestinal tract of human and other animals (53). *Desulfovibrio* has been controversially proposed as either commensal or detrimental (54). The results of correlation analysis indicated that dietary AOE might modulate the flavor amino acids deposition in the muscle by altering the microbial composition.

## Conclusions

Dietary supplementation with 30 mg/kg AOE modulated the intestinal microbial composition and maintained intestine integrity. It also increased meat nutrition profile and flavor through promoting total, essential, and flavor amino acids deposition in breast muscle. AOE as an antibiotic alternative displayed the potential in maintaining intestinal health and improving meat quality.

## Data Availability Statement

The datasets presented in this study can be found in online repositories. The names of the repository/repositories and accession number(s) can be found in below: NCBI (accession: PRJNA755524).

## Ethics Statement

The animal study was reviewed and approved by Animal Care and Use of Chinese Academy of Tropical Agricultural Sciences (Haikou, Hainan Province, China).

## Author Contributions

FJ, LG, and TX contributed to the study design, conducted the animal experiments, and wrote the manuscript. GR, CH, WS, DW, WP, and HW executed the lab analysis. DL and QL performed the statistical analysis. HD and HZ revised the paper. All authors contributed to the article and approved the submitted version.

## Funding

The present work was jointly supported by China Agriculture Research System of MOF and MARA, Hainan Provincial Natural Science Foundation of China (321MS087), Central Public-interest Scientific Institution Basal Research Fund for Chinese Academy of Tropical Agricultural Sciences (1630032017034, 1630032021004), and National Natural Science Foundation of China (31972553).

## Conflict of Interest

The authors declare that the research was conducted in the absence of any commercial or financial relationships that could be construed as a potential conflict of interest.

## Publisher's Note

All claims expressed in this article are solely those of the authors and do not necessarily represent those of their affiliated organizations, or those of the publisher, the editors and the reviewers. Any product that may be evaluated in this article, or claim that may be made by its manufacturer, is not guaranteed or endorsed by the publisher.
